# Evaluation of the Protein Content and Variability of Olluco (*Ullucus tuberosus* Caldas): Implications for Its Revaluation as an Andean Food of High Nutritional Value

**DOI:** 10.1155/tswj/9075681

**Published:** 2025-11-02

**Authors:** Narcizo Gómez-Villanes, Rita Girón-Aguilar, Vidal Aquino-Zacarías, Mario Monteghirfo-Gomero, María Custodio, Kevin Ortega-Quispe, Dennis Ccopi-Trucios, Samuel Pizarro-Carcausto

**Affiliations:** ^1^Faculty of Agronomy, Universidad Nacional del Centro del Perú, Jauja, Peru; ^2^Santa Ana Agricultural Experimental Station, Directorate of Genetic Resources and Biotechnology, National Institute for Agrarian Innovation, Huancayo, Peru; ^3^Faculty of Medicine, Universidad Nacional Mayor de San Marcos, Lima, Peru; ^4^Faculty of Human Medicine, Universidad Nacional del Centro del Perú, Huancayo, Peru; ^5^Santa Ana Agricultural Experimental Station, Direction of Supervision and Monitoring in the Agricultural Experimental Stations, National Institute of Agrarian Innovation, Huancayo, Peru

**Keywords:** Andean tuber, electrophoresis, Kjeldahl method, nutritional value, olluco, protein variability

## Abstract

Olluco (*Ullucus tuberosus* Caldas) is an Andean tuber essential in the diet of communities in the Andean regions of South America, generally cultivated at altitudes above 2800 m above sea level. Despite its importance, information on its nutritional composition, particularly protein variability among varieties, is limited. This study determined the total protein content of 50 freeze-dried olluco varieties by the semimicro Kjeldahl method and evaluated the variability of the protein profile of these varieties by electrophoresis techniques (SDS-PAGE and ND-PAGE). The results revealed a wide range of protein content: 20% of the varieties showed high content (10.07–11.55 g/100 g dry weight), 62% medium content (7.00–9.98 g/100 g dry weight), and 18% low content (5.60–6.65 g/100 g dry weight). Analysis of protein profile variability revealed a remarkable similarity in the low molecular weight proteins, especially the intense bands close to 14 and 24 kDa. Cluster analysis of protein patterns obtained by SDS-PAGE allowed us to establish genetic distances and phylogenetic relationships between the varieties studied. The variability in the protein content of olluco suggests that the region of origin may influence the nutritional composition of the varieties. These findings highlight the protein variability of olluco and its potential as a valuable genetic resource for food security and nutrition in the Andean region.

## 1. Introduction

The Andean region is characterized by its great diversity of roots and tubers, which constitute a staple food for the rural population due to their high carbohydrate content, especially starch. Olluco (*Ullucus tuberosus* Caldas), an Andean tuber from the Basellaceae family, is a staple in the local diet due to its high carbohydrate content [1, 2]. This crop, domesticated since pre-Hispanic times, approximately 5500 years ago [3–5], adapts to various altitudes [6, 7]. In the South American Andes, olluco is the second most cultivated and economically important tuber, being cultivated from Venezuela to northwestern Argentina, covering countries such as Colombia, Ecuador, Peru, and Bolivia [8]. The erect stems of the olluco plant reach heights of 30–80 cm. Its tubers, fleshy and veiny, show round, cylindrical, or twisted shapes, with diameters of 1–6 cm and a smooth, shiny surface. The skin of the tubers shows a diversity of colors, ranging from yellowish white to magenta, including a wide range of intermediate shades [9].

Olluco stands out for its rich chemical composition, being an excellent source of soluble carbohydrates in its dry matter. The composition of these compounds varies significantly between fresh and dry tubers. Fresh tubers have a high moisture content, ranging from 83.7% to 85.9%, and a starch content of 14%. The variability in the chemical composition of olluco is wide and depends on factors such as variety and area of cultivation. Protein content varies between 10.8% and 15.7% on a dry basis [10, 11]. Olluco provides a nutritional energy value of 4.05–4.44 Kcal/g, which makes it a valuable nutritional source, especially for its protein content. In addition to its nutritional value, olluco is known for its medicinal properties. Traditionally, it is used to treat burns and prevent scarring, and there is local evidence supporting its efficacy and the presence of bioactive compounds [12]. Despite its historical and nutritional importance, there is a significant gap in understanding its protein composition and genetic variability among its various varieties, limiting its full potential as a source of food with high nutritional value.

The study of genetic variation in olluco is essential to understanding its biological diversity, since analysis of its protein composition directly reflects differences in its genetic code [13, 14]. Previous studies, for example, have identified two groups of major protein bands in the ranges of 6.5–12 kDa and 25–58 kDa using pore gradient electrophoresis [14]. In addition, 21 bands were identified in unreduced samples and 25 in reduced samples using discontinuous and pore gradient electrophoresis with SDS [15]. Unlike these studies, this study seeks to fill that gap through a comprehensive assessment of total protein content and characterization of the protein profile of 50 varieties of olluco. To achieve this, electrophoresis techniques (sodium dodecyl sulfate polyacrylamide gel electrophoresis [SDS-PAGE] and nondenaturing polyacrylamide gel electrophoresis [ND-PAGE]) are combined to provide a more comprehensive view of genetic variability. The findings of this research have the potential to revalue olluco as an Andean food with high nutritional value, laying the scientific foundation for future programs to improve and conserve this valuable crop.

## 2. Materials and Methods

### 2.1. Samples and Reagents

**Figure 1:**
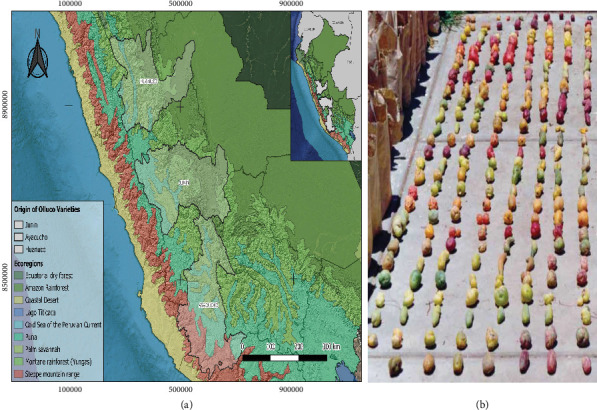
Variability of the 50 varieties of olluco. (a) Region of origin of olluco varieties in the central Peruvian highlands. (b) Germplasm bank of *Ullucus tuberosus* Caldas.

Olluco samples (50 varieties) were obtained from the Germplasm Bank of the National Institute of Agrarian Research (INIA), specifically from the National Program for Research in Genetic Resources and Biotechnology (PRONIRGEB), located at the Santa Ana Experimental Station, Huancayo (3200 masl.). The varieties have a localized origin in the regions of Huánuco, Junín, and Ayacucho according to INIA records (Figure 1). Samples were selected and transported for evaluation. In the laboratory, the samples were washed with distilled water, completely lyophilized, and stored at −-20°C until analysis.

### 2.2. Determination of Total Nitrogen by Semimicro Kjeldahl Method

The determination of total nitrogen was carried out by the semimicro Kjeldahl method [16]. For this purpose, 0.5 g of freeze-dried samples of each olluco variety were weighed and transferred to digestion cuvettes. Each sample was added to a mixture of 2 g of salt and 4 mL of concentrated sulfuric acid. The samples were heated at 460°C for 2 h, and the digested samples were diluted with 15 mL of distilled water. Subsequently, distillation was carried out using a semimicro Kjeldahl distiller. The ammonium sulfate present in the digested sample was treated with 15 mL of 50% sodium hydroxide, releasing ammonia. The released ammonia was collected in 10 mL of 2% boric acid with methyl red indicator. Finally, titration was carried out in a 100 mL beaker, using a burette and 0.1 N hydrochloric acid as titrant. To determine the protein content of the olluco, a conversion factor of 6.25 was applied to the total nitrogen obtained.

### 2.3. Total Protein Extraction

For olluco total protein extraction, tubers were frozen at −20°C for 24 h. After freezing, they were weighed to determine the amount of antioxidant solution required (500 *μ*L/100 g of tuber). The tubers were thawed and peeled. Subsequently, a juice extractor was used to obtain the extract. To prevent protein oxidation, an antioxidant solution composed of 20% sodium sulfite and 15% sodium bisulfite was added until the required final concentration was reached. The extract obtained was centrifuged at 3000 rpm for 10 min to remove starch and other residues. The supernatant was kept refrigerated at −20°C until further analysis [17, 18].

### 2.4. Protein Electrophoresis

#### 2.4.1. SDS-PAGE

##### 2.4.1.1. Sample Preparation

One milliliter of olluco protein extract was taken and precipitated with 4 volumes of cold acetone. The mixture was centrifuged at 3000 rpm for 5 min, the supernatant was discarded, and the sediment was resuspended in 250 *μ*L of a 1:1 solution of sample buffer and water. The sample buffer contained 0.5 M Tris-HCl (pH 6.8), 10% SDS, 20% glycerol, and 10% 2-mercaptoethanol according to the batch system described by Laemmli [19]. The sample was homogenized, centrifuged at 12,000 rpm for 5 min, and heated in a water bath at 95°C for 3 min. After cooling the sample with water, 5 *μ*L of amido black.

##### 2.4.1.2. Gel Preparation

Polyacrylamide gels at 12% (separating gel) and 4% (stacking gel) were prepared on a vertical gel plate of 1 mm thickness [20]. The protocol for preparing SDS-PAGE gels consisted of preparing the separating gel by mixing a solution of acrylamide/bisacrylamide, Tris-HCl at pH 8.9, 10% ammonium persulfate (APS), and distilled water. After degassing the mixture, TEMED was added to initiate polymerization. The solution is poured between the glass plates and allowed to polymerize for 30–40 min. Next, the stacking gel is prepared with a similar mixture, but with different concentrations of acrylamide/bisacrylamide and Tris-HCl at pH 6.7. After degassing and adding TEMED, this solution is poured onto the already polymerized separating gel. Finally, a Teflon comb is inserted and the stacking gel is allowed to polymerize for 30 min, thus completing the assembly of the gel for electrophoresis (Table 1).

##### 2.4.1.3. Electrophoresis and Staining

The comb was removed, and 25 *μ*L of each sample was loaded into the wells, including one lane with a molecular weight standard (14.2–66 kDa, Sigma). Electrophoresis was performed in a vertical chamber for 6 h, applying a constant current of 10–40 mA. Gels were stained overnight with a solution of Coomassie blue R-250 (1% in 40 mL acetic acid, 12 mL 10% trichloroacetic acid, 40 mL methanol, and 160 mL distilled water). Decolorization was performed with a solution of 10 mL acetic acid, 60 mL methanol, and 140 mL distilled water.

#### 2.4.2. ND-PAGE

For ND-PAGE, total olluco proteins were treated with a 60% sucrose solution to increase the sample density and 0.003% amido black as a mobility indicator. The discontinuous system was used, with a 12% separating gel (pH 8.8) and a 5% stacking gel (pH 6.8). Electrophoresis, staining, and decolorization of the gels were performed following the same procedure described for SDS-PAGE [21].

### 2.5. Data Analysis

SDS-PAGE and ND-PAGE gels were photographed and analyzed to determine protein banding patterns. The unweighted pair-group method with arithmetic means (UPGMA) was used to evaluate the genetic similarity between olluco varieties. The UPGMA analysis was performed using PyElph 1.4 software, which generated a dendrogram revealing the genetic relationships between the varieties. The dendrogram was interpreted by considering the length of the branches and the formation of groups or clusters [22]. Varieties that were grouped in the same cluster were considered genetically more similar based on their protein patterns. In addition to the UPGMA analysis, a visual analysis of protein band patterns was performed to identify qualitative and quantitative differences between varieties. The number, intensity, and position of the protease bands were compared to determine the variability of proteins among olluco varieties.

## 3. Results and Discussion

### 3.1. Total Protein Content in Different Varieties of Freeze-Dried Olluco

Table 2 shows the protein content of 50 varieties of olluco (*Ullucus tuberosus* Caldas) from the Peruvian regions of Huánuco, Huancayo, and Ayacucho. There was significant variation in protein content between varieties, with values ranging from 5.60 g/100 g dry weight (variety SA-042) to 11.55 g/100 g dry weight (variety HNCO-062). The Ayacucho (AYA) variety recorded the highest average protein content (9.45 g/100 g dry weight), with the lowest dispersion, as evidenced by a standard deviation (SD) of 0.95 and a coefficient of variation (CV) of 10.03%. These data suggest homogeneity and stability in protein expression within these varieties. In contrast, the Huánuco (HNCO) and Huancayo (SA) varieties showed greater variability, with averages of 8.58 g/100 g dry weight (SD = 1.62; CV = 18.89%) and 8.20 g/100 g dry weight (SD = 1.58; CV = 19.25%), respectively (Table 3).

**Figure 2:**
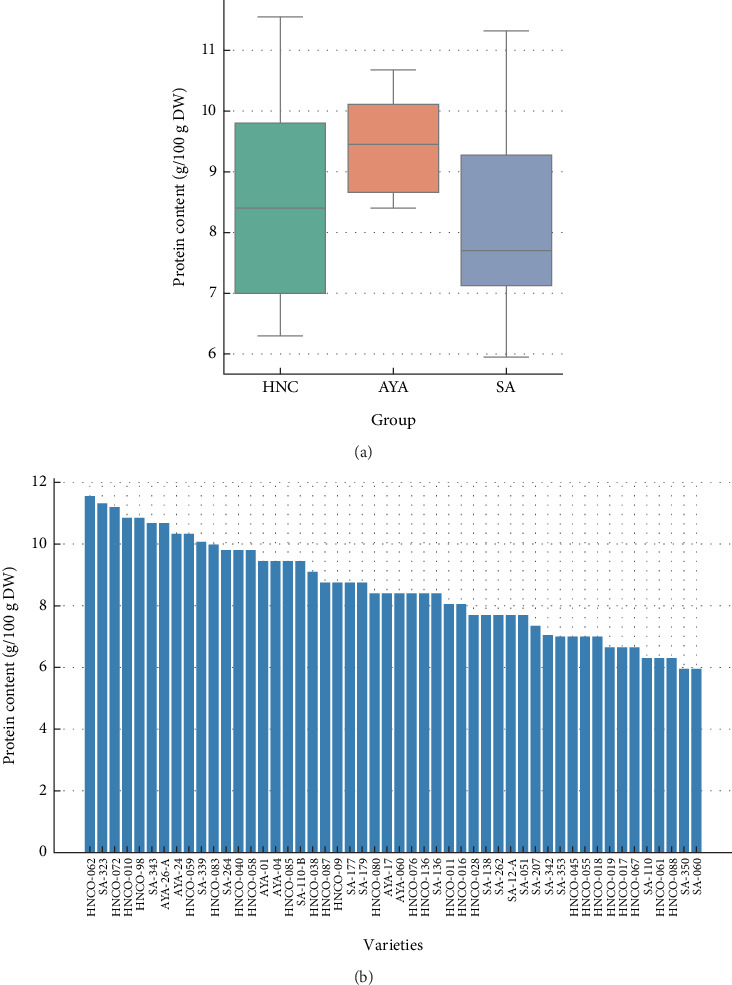
Comparison and distribution of protein content in olluco varieties according to the region of origin. (a) Box plot of protein content among olluco varieties (HNCO, AYA, and SA). (b) Individual distribution of protein content for each of the 50 varieties ordered from highest to lowest.

Figure 2a shows the comparison of protein content between the three groups evaluated using box plots. The AYA variety had the highest average value (9.45 g/100 g dry weight), with a compact and stable distribution. This narrow range of variation, which ranged from 8.40 to 10.68 g/100 g dry weight, indicates remarkable homogeneity and stability in protein expression within these varieties. In contrast, the HNCO variety recorded an average of 8.58 g/100 g dry weight with a wider range (6.30–11.55 g/100 g dry weight), reflecting greater data dispersion. Similarly, the SA variety showed a lower average (8.20 g/100 g dry weight) and the widest range (5.95–11.32 g/100 g dry weight). This indicates wide variability in the protein content of the varieties, with genotypes presenting both extremely high and low values.

Figure 2b shows the ranked distribution of protein content for each of the 50 varieties. At the upper end of the range, the varieties HNCO-062 (11.55 g/100 g dry weight), SA-323 (11.32 g/100 g dry weight), and HNCO-072 (11.20 g/100 g dry weight) stood out, exceeding 11 g/100 g dry weight. Other varieties with high protein content were HNCO-010 (10.85 g/100 g dry weight) and AYA-26-A (10.68 g/100 g dry weight). At the lower end, the varieties SA-350 and SA-060 had the lowest values (5.95 g/100 g dry weight), followed by HNCO-061 (6.30 g/100 g dry weight) and SA-110 (6.30 g/100 g dry weight). This range in the data revealed a difference of almost 6 g/100 g between the varieties with the highest and lowest protein content, demonstrating the high genetic variability present in the material analyzed.

This diversity reveals the genetic richness of olluco and its potential as a source of nutrients. The results also show that some varieties from Huánuco and Huancayo tend to have a higher protein content compared to those from Ayacucho. However, protein levels in olluco varieties from Ayacucho are also high. This diversity highlights the genetic richness of olluco and its potential as a source of nutrients. These results are consistent with previous studies, such as those of Camarena [23] and Sanchez-Portillo et al. [24], which also report a wide variability in the protein content of olluco, with ranges similar to those found in this study. However, it is important to consider that differences in the varieties studied, growing conditions, and analysis methodologies could explain the variations in the specific values reported.

The presence of high-protein varieties suggests the potential of olluco as a valuable source of dietary protein, especially in Andean communities where protein availability may be limited. This aligns with research that highlights olluco as a key resource for food security in the Andes due to its adaptability and nutritional value [25]. In addition, studies on the amino acid composition of olluco have shown its potential as a source of essential amino acids, reinforcing its nutritional value [26]. Research on the antioxidative properties and bioactive compounds of olluco also supports its potential as a functional food [27]. Finally, studies on olluco revaluation strategies emphasize the importance of selecting varieties with high nutritional value to promote its cultivation and consumption [11].

**Figure 3:**
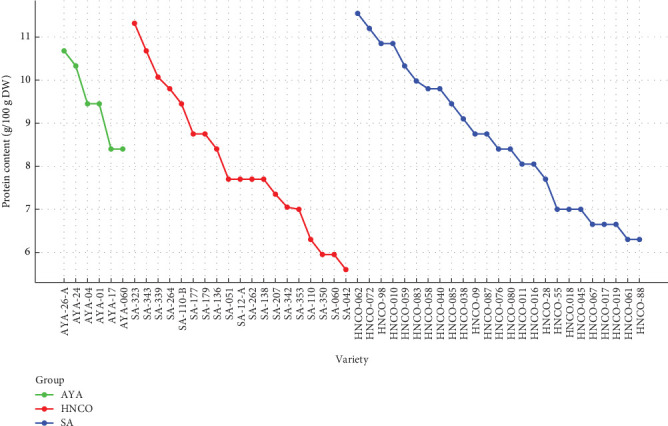
Variability of the protein content in 50 varieties of olluco.

Figure 3 shows the distribution of protein content in olluco varieties from HNCO (blue), AYA (green), and SA (red). A clear differentiation between regions was observed, highlighting the influence of agroecological conditions on the protein composition of the varieties. Varieties from HNCO show a wide distribution of protein content, with a predominant range between 8.00 and 10.85 g/100 g, and some varieties reaching high levels of up to 11.55 g/100 g. This indicates that the conditions of this region favor the development of varieties with a relatively high protein content. On the other hand, the varieties from Ayacucho show a smaller dispersion, with values ranging from 8.40 to 10.68 g/100 g. This group maintains a moderately high protein level, which could be related to the particular climatic and edaphic conditions of the area. The SA varieties cover a wider range of protein content from relatively low values (5.95 g/100 g) to some of the highest observed (11.32 g/100 g). This variability suggests that the varieties grown in Huancayo show greater genetic heterogeneity or that they have been subjected to different agronomic management conditions. The predominance of the medium range of protein content (7.00–9.98 g/100 g) in all regions highlights the adaptability of the crop to different Andean environments. However, the presence of varieties with high protein levels in HNCO and SA suggests an important potential for genetic improvement and selection of crops with higher nutritional value. Data from Collazos et al. [28] corroborate this statement. They also emphasize nutritional variability when samples are dehydrated.

The nonuniform distribution of protein content, evidenced by the difference in the height of the bars, underlines the genetic variability and the influence of external factors on the composition of olluco. This figure highlights the importance of olluco in Andean nutrition, especially the varieties with medium and high protein content, and raises the need for further studies to fully understand the factors that modulate protein variability and thus optimize its nutritional value.

### 3.2. Analysis of the Variability of the Protein Profile of Olluco Varieties Using Electrophoresis

**Figure 4:**
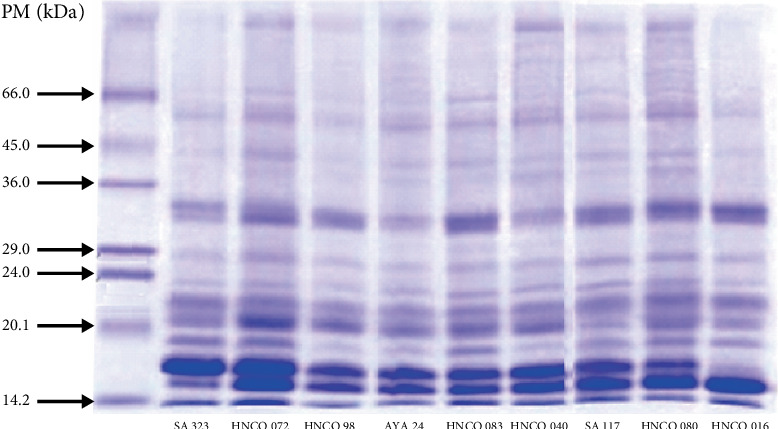
SDS-PAGE electrophoresis (12%) of total proteins of nine promising olluco varieties.

Figure 4 shows the electrophoretic patterns (SDS-PAGE) of the total proteins of nine olluco varieties, revealing a remarkable similarity in the low molecular weight proteins, especially the intense bands close to 14 and 24 kDa. These bands, the most abundant, suggest a common genetic basis among the varieties, possibly indicating the conservation of essential proteins throughout the evolution of olluco. However, differences are observed in the medium and high molecular weight proteins (33–80 kDa), with distinct bands between 29 and 36 kDa, suggesting greater genetic variability in these regions. These results are in agreement with previous studies that highlight the importance of olluco genetic diversity for its adaptability to diverse environments [26]. The variability observed in medium and high molecular weight proteins could be related to adaptation to different environmental and cultivation conditions, or to specific characteristics of each variety. The banding patterns obtained by SDS-PAGE could be useful as molecular markers to differentiate varieties and evaluate genetic diversity in breeding and conservation programs.

In addition to their usefulness as molecular markers, the banding patterns obtained by SDS-PAGE could provide valuable information on the protein composition and nutritional value of olluco varieties. Future studies could focus on the identification of the specific proteins present in the distinctive bands using techniques such as mass spectrometry. This information would allow a better understanding of the function of these proteins and their relationship with the agronomic and nutritional characteristics of olluco. Comparative studies with other Andean tuber species could also be carried out to identify conserved or species-specific proteins, which would contribute to the understanding of the evolution and adaptation of these crops. The identification of varieties with unique protein profiles could be very useful for breeding programs, allowing the selection of varieties with desirable characteristics, such as high protein content or disease resistance. Ultimately, these studies could contribute to the revaluation of olluco as an Andean crop of high nutritional value and its conservation as an important genetic resource.

**Figure 5:**
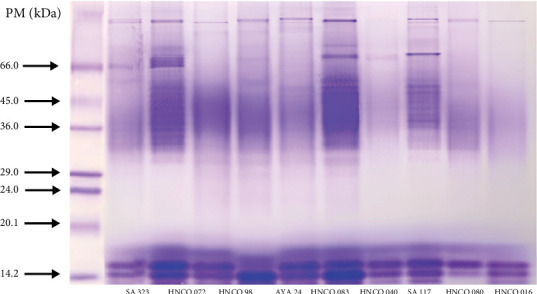
Nondenaturing polyacrylamide gel electrophoresis (ND-PAGE) of native olluco proteins.

Figure 5 presents the ND-PAGE patterns of total native proteins extracted from 10 promising olluco varieties. Visualization reveals two distinctive protein groups: a set of medium- and high-molecular-weight proteins that exhibit a characteristic banding pattern and a group of low-molecular-weight proteins that reveal a pattern very similar to that observed in denaturing gels (SDS-PAGE). This similarity in the low-molecular-weight proteins suggests that these proteins migrate mainly as a function of their size, independent of denaturing conditions, which could indicate a high structural stability. The presence of variations in protein profiles, especially in those of medium and high molecular weight, could reflect differences in the amino acid sequence, which in turn suggests allelic variations in the genes encoding these proteins.

Comparison with previous studies, such as those conducted by Monteghirfo and Yarleque-Chocas [29] in maca and Shah et al. [15] in olluco tubers, reveals similar patterns in protein distribution by molecular weight. These similarities could indicate evolutionary links or common biochemical adaptations between these species despite their genetic differences [30]. The results obtained are in agreement with studies that highlight the importance of genetic variability detected by electrophoresis in the adaptability of olluco to different ecological niches [31]. In addition, the observed protein diversity could serve as a useful marker to identify specific cultivars or varieties of olluco, opening an interesting field of research on the genetic regulation of proteins in this species and their relationship with environmental and genetic factors.

The observation of different protein patterns among olluco varieties reinforces the idea that variability in protein profiles could be due to variations in the amino acid sequence, which implies the existence of allelic variations in the genes encoding these proteins. This finding opens an interesting field of research on the genetic regulation of proteins in olluco and their relationship with environmental and genetic factors. The observed protein diversity could serve as a useful marker to identify specific cultivars or varieties of olluco, which would have significant implications for the conservation of genetic diversity and the improvement of this important Andean crop.

**Figure 6:**
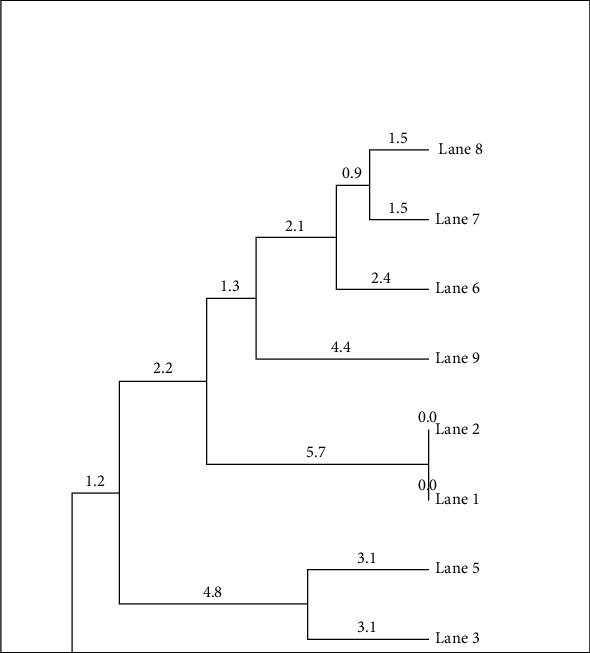
UPGMA grouping of olluco varieties based on protein profiling (SDS-PAGE).

Figure 6 presents a dendrogram generated by the UPGMA method based on the protein profiles obtained by SDS-PAGE of nine promising olluco varieties. This analysis, performed with PyElph software, made it possible to establish the genetic distances and phylogenetic relationships among the varieties studied. The formation of four main groups in the dendrogram indicates significant genetic diversity among the varieties, suggesting the existence of differentiated phylogenetic relationships. The numerical distances in the branches of the dendrogram reflect the degree of genetic divergence between the varieties, allowing the identification of those most related and those most distant. It can be observed, for example, that the varieties “Lane 1” and “Lane 2” form a very close cluster (distance of 0.0), suggesting a high similarity in their protein profile despite possible differences in their geographical origin. The existence of distinctive groups, such as that formed by “Lane 7” and “Lane 8,” which are separated from the rest by a greater distance (1.5), highlights the intrinsic genetic variability of the species. These findings are supported by the study by García-Díaz et al. [32], which reveals marked genetic differentiation between olluco populations across Peru's diverse agroecological zones, suggesting that olluco diversity is much greater and strongly influenced by geographic and environmental factors. Other research also demonstrates the need to include varieties from different regions in order to accurately identify true patterns of diversity [11].

The variability in protein content found in this study, reflected in the dendrogram distances, suggests that the region of origin may influence the nutritional composition of varieties. For example, in the case of quinoa, studies have shown that its nutritional composition and gene expression vary significantly depending on the environmental and geographical conditions of cultivation [33]. Similarly, factors such as soil type, altitude, and local cultivation practices likely contribute to the differences observed in protein content among the varieties analyzed. These findings have significant implications for breeding and conservation programs for Andean genetic resources such as olluco. The genetic diversity observed is a fundamental pillar for the development of more efficient breeding strategies as it allows for the selection of genotypes with superior characteristics. This not only helps improve crop productivity and quality but is also vital for strengthening their resilience to environmental challenges, such as climate change and the emergence of new pests and diseases, as indicated by previous studies [34]. Preserving this genetic variability is therefore a key measure for ensuring the long-term sustainability of these crops.

To maximize the benefits of this genetic diversity, it is essential to integrate ancestral knowledge and traditional agricultural practices into conservation and improvement programs. Collaboration with local communities and adapting programs to their specific needs, as suggested by research [35], is essential for success. This approach not only helps to maintain the gene pool but also facilitates the creation of new cultivars with desirable characteristics, such as greater resistance or nutritional value. In this way, genetic erosion is actively combated, a phenomenon that could severely compromise future breeding efforts and jeopardize food security, as documented in the scientific literature [36]. Therefore, fostering genetic diversity is vital to improving the productivity of olluco cultivars and their resilience to disease and environmental fluctuations. Identifying groups of genetically similar or distantly related varieties can guide the selection of parents for crosses and the development of new varieties with improved agronomic and nutritional characteristics [37]. By combining genotypes that possess complementary traits, such as high resistance to pathogens and excellent nutritional qualities, the development of new improved varieties is accelerated [38]. This methodology not only optimizes crop yield but also ensures that future generations of olluco have the potential to withstand any agronomic challenges that may arise. Conserving the genetic diversity of olluco is crucial to ensuring the availability of genetic resources for future generations and maintaining food security in the Andean region. When local varieties are lost, a process known as genetic erosion, the genetic material available for research and improvement is reduced. This not only jeopardizes the crop's ability to adapt but also threatens the stability of food systems. Therefore, every action to conserve olluco, whether in the field or in germplasm banks, builds a genetic legacy that will be crucial for addressing the nutritional and environmental challenges of the future.

### 3.3. Future Perspectives

This study not only lays the foundation for understanding protein diversity in olluco varieties but also opens numerous avenues for future research. One promising approach would be the identification and characterization of the specific proteins responsible for the observed differences among varieties. Mass spectrometry and other proteomic techniques could be employed for this purpose, making it possible to determine the function of these proteins and their relationship with agronomic and nutritional characteristics. In addition, gene sequencing studies and gene expression analysis could reveal the molecular mechanisms that regulate the synthesis of these proteins. The information obtained could be used to develop more precise and efficient molecular markers for the selection of superior varieties in breeding programs.

### 3.4. Limitations of the Study

It is important to recognize the limitations of this study. First, the sample size of olluco varieties analyzed may not fully represent the genetic diversity of the species. The inclusion of a larger number of varieties from different geographic regions would broaden the scope of the results and provide a more complete picture of the protein diversity of olluco. Second, the study focused on the analysis of total proteins extracted from tubers, but protein variability in other plant organs, such as leaves or roots, was not explored. The inclusion of other organs could reveal additional differences in protein profiles and provide valuable information on olluco physiology and metabolism.

### 3.5. Implications for Olluco Revaluation

This study highlights the importance of protein diversity in olluco as a key factor for its revaluation as an Andean food of high nutritional value. The identification of varieties with unique and valuable protein profiles, including those with high content of essential amino acids, opens new possibilities for the development of innovative and nutritious food products. Promoting the consumption of these varieties could contribute to improving food security and nutrition in the Andean region, especially in communities where the availability of high-quality protein is limited. In addition, the genetic diversity observed in olluco suggests that this crop has great potential to adapt to different environmental and growing conditions, which is crucial in the context of climate change. The conservation of this genetic diversity is therefore fundamental to ensure the resilience of olluco and its contribution to long-term food security.

In addition, the results of this study have implications for the genetic improvement of olluco and the development of new food products. The identification of molecular markers based on protein profiles could facilitate the selection of varieties with desirable traits, such as disease resistance or higher nutrient content. Likewise, understanding the function of specific proteins present in olluco could open new avenues for the development of functional foods and nutraceuticals. The revaluation of olluco as an Andean crop of high nutritional value could have a positive impact on the local economy and on the promotion of sustainable agricultural practices that preserve the region's biodiversity.

## 4. Conclusions

This study reveals significant variability in total protein content among the freeze-dried olluco varieties analyzed, which highlights the genetic richness of this Andean crop. The diversity observed in the protein profiles by SDS-PAGE suggests that there are significant differences in protein composition among varieties, which could be related to their adaptation to different environmental and cultivation conditions. The identification of varieties with high protein content and unique protein profiles has important implications for the revaluation of olluco as an Andean food of high nutritional value and for its use in breeding programs.

The results obtained by UPGMA clustering analysis, based on protein profiles, confirmed the genetic diversity among olluco varieties and revealed the existence of distinct phylogenetic relationships. The formation of distinct clusters in the dendrogram suggests that there are distinct genetic lineages within the species, which could be of great utility for the selection of varieties with desirable traits. The identification of molecular markers based on protein profiles could facilitate the selection of superior varieties in breeding programs and contribute to the conservation of olluco genetic diversity. Taken together, these findings highlight the potential of olluco as a valuable genetic resource for food security and nutrition in the Andean region.

## Figures and Tables

**Table 1:** Composition and running conditions for SDS-PAGE and ND-PAGE.

**Protein electrophoresis**	**Buffers**	**Composition**	**pH**	**Amperes (A)**	**Duration (hours)**
SDS-PAGE	Sample buffer	Tris-HCl 0.5 M, 10% of SDS, 20% of glycerol, and 2% of 2-mercaptoetanol	6.8		
	Separating gel	0.33 volumes of acrylamide/bisacrylamide solution (30% acrylamide), 0.2 volumes of 0.5 M Tris-HCl, 0.2 volumes of 1.5 M Tris-HCl, 0.003 volumes of 10% ammonium persulfate (APS), and distilled water. The undegassed mixture was added to TEMED	8.9		
	Stacking gel	0.2 volumes of acrylamide/bisacrylamide solution (20% acrylamide), 0.2 volumes of 0.25 M Tris-HCl, 0.25 volumes of 0.5 M Tris-HCl, 0.004 volumes of 10% APS, and distilled water	6.7		
	Running buffer	25 mM Tris-HCl, 192 mM glycine, and 0.1% SDS	8.3	10 mA	6
	Staining buffer	1% Coomassie blue R-250 staining solution in 45 mL acetic acid, 12 mL trichloroacetic acid, 40 mL methanol, and 160 mL distilled water		40	12

ND-PAGE	Sample processing	Total proteins were treated with a 60% sucrose solution to increase sample density and with 0.003% bromophenol blue as a mobility indicator			
	Stacking gel	0.2 volumes of acrylamide/bisacrylamide solution (20% acrylamide), 0.25 volumes of 0.5 M Tris-HCl, 0.004 volumes of 10% APS, and distilled water were mixed	6.8		
	Separating gel	0.33 volumes of acrylamide/bisacrylamide solution (30% acrylamide), 0.2 volumes of 0.5 M Tris-HCl, 0.2 volumes of 1.5 M Tris-HCl, 0.003 volumes of 10% ammonium persulfate (APS), and distilled water were added. TEMED was added to the mixture.	8.8		
	Running buffer	25 mM Tris-HCl, 192 mM glycine, and 0.1% SDS	8.3	10 mA	6
	Staining buffer	1% Coomassie blue R-250 staining solution in 45 mL acetic acid, 12 mL trichloroacetic acid, 40 mL methanol, and 160 mL distilled water		40	12

Abbreviation: mA, milliamperes.

**Table 2:** Mean protein content (grams per 100 g dry weight) in 50 varieties of *Ullucus tuberosus* Caldas according to the region of origin.

**Region of origin**	**Variety**	**Mean protein content**	**Variety**	**Mean protein content**
Huánuco	HNCO-062	11.55	HNCO-080	8.40
	HNCO-072	11.20	HNCO-011	8.05
	HNCO-98	10.85	HNCO-016	8.05
	HNCO-010	10.85	HNCO-28	7.70
	HNCO-059	10.33	HNCO-55	7.00
	HNCO-083	9.98	HNCO.018	7.00
	HNCO-058	9.80	HNCO-045	7.00
	HNCO-040	9.80	HNCO-067	6.65
	HNCO-085	9.45	HNCO-017	6.65
	HNCO-038	9.10	HNCO-019	6.65
	HNCO-09	8.75	HNCO-061	6.30
	HNCO-087	8.75	HNCO-88	6.30
	HNCO-076	8.40		

Huancayo	SA-323	11.32	SA-262	7.70
	SA-343	10.68	SA-138	7.70
	SA-339	10.07	SA-207	7.35
	SA-264	9.80	SA-342	7.05
	SA-110-B	9.45	SA-353	7.00
	SA-177	8.75	SA-110	6.30
	SA-179	8.75	SA-350	5.95
	SA-136	8.40	SA-060	5.95
	SA-051	7.70	SA-042	5.60
	SA-12-A	7.70		

Ayacucho	AYA-26-A	10.68	AYA-01	9.45
	AYA-24	10.33	AYA-17	8.40
	AYA-04	9.45	AYA-060	8.40

**Table 3:** Comparative analysis of protein content in varieties of *Ullucus tuberosus* Caldas from different regions of Peru.

**Variety**	**N**	**Mean**	**SD**	**Minimum**	**Median**	**Maximum**	**CV (%)**
AYA	6	9.45	0.95	8.40	9.45	10.68	10.03
HNCO	26	8.58	1.62	6.30	8.40	11.55	18.89
SA	18	8.20	1.58	5.95	7.70	11.32	19.25

Abbreviations: CV, coefficient of variation; SD, standard deviation.

## Data Availability

Data supporting the results of this study are available from the corresponding author upon request.
